# Impact of Human Dermal Microvascular Endothelial Cells on Primary Dermal Fibroblasts in Response to Inflammatory Stress

**DOI:** 10.3389/fcell.2019.00044

**Published:** 2019-04-03

**Authors:** Benjamin Sanchez, Linan Li, Joshua Dulong, Géraldine Aimond, Jérôme Lamartine, Guangrong Liu, Dominique Sigaudo-Roussel

**Affiliations:** ^1^CNRS UMR 5305, Laboratoire de Biologie Tissulaire et Ingénierie Thérapeutique, Lyon, France; ^2^Claude Bernard University Lyon 1, Villeurbanne, France; ^3^Infinitus Company Ltd., Guangzhou, China

**Keywords:** fibroblasts, endothelial cells, inflammation, extracellular matrix, collagen, elastin

## Abstract

The aim of the present study was to evaluate the impact of the microenvironment produced by dermal microvascular endothelial cells, secondary to a pro-inflammatory challenge, on 2D culture models using dermal fibroblasts and in 3D reconstructed skin model using dermal fibroblasts and keratinocytes from healthy donors. We hypothesized that specific microvascular endothelial low grade inflammation could change fibroblasts phenotype and be involved in extracellular matrix (ECM) modification and skin alteration. Following IFNγ, TNFα, IL-1β pro-inflammatory stress on Human Dermal Endothelial Cells (HDMEC) we observed the increased release of Chemokine ligand 2 (CCL2), IL-6 and IL-8 but not VEGF-A in the conditioned medium (CM). The subsequent addition of this endothelial pro-inflammatory CM in dermal fibroblasts revealed an upregulation of *IL6*, *IL8* and *CCL2* but no *NF-κB* gene expression. The resulting ECM formation was impaired with a reduction of the collagen 1 network and a decrease in *COL1A1* gene expression in 2D and 3D models. Collagen 1 and pro-LOX protein expression were significantly reduced confirming an impairment of the collagen network related to endothelial inflammation secretion. To conclude, this work showed that, without any immune cells, the endothelial secretion in response to a pro-inflammatory stress is able to activate the fibroblasts that will maintain the pro-inflammatory environment and exacerbate ECM degradation.

## Introduction

Skin microcirculation is located deep in the dermis layer up to the reticular dermis close to the epidermis. Skin microcirculation plays a significant role in regulating skin homeostasis, thermoregulation, blood pressure and inflammatory response (Charkoudian, 2003; Gutterman et al., 2016). It also allows nutrients and systemic factors to reach the whole skin tissue throughout the skin microvascular network from arteries to small capillaries close to the epidermis. Microvascular endothelial cells are the major components of dermal blood vessels and are clearly involved in skin inflammatory process. Endothelial cells participate to synthesize and secrete chemokine and cytokine and are implicated in recruiting immune cells in response to dysfunctions due to different factors (e.g., UV, heat, pathological and physiological processes) (Swerlick and Lawley, 1993).

Specific inflammatory mediators are described as endothelium activators like IL-1β or TNF-α, permitting vessels hyper-permeability allowing leucocyte infiltration. These vascular modulations are involved in skin diseases linked to a chronic inflammation (McGee et al., 1995; Huggenberger and Detmar, 2011). *In vitro*, TNF-α, IL-1β, and IFN-γ may alter vascular processes and cells behaviors, inducing inflammation stress. A mix of these cytokines induces synergistic effect producing inflammatory responses, which could induce at low concentration expression of chemokine like CCL2 or RANTES implicated in vessels permeability on endothelial cells (Goebeler et al., 1997).

Thus, inflammation is a common condition that affects the vasculature, but is also a skin feature that occurs in long-term pathologies, as well as in short-term conditions at high or low-grade level (Huggenberger and Detmar, 2011). Dermal fibroblasts are the key cells to synthetize the ECM composed by many components including collagen and ELN fibers. Fibroblasts are also known to stimulate vascular inflammation through cytokines and chemokines production (Singer and Clark, 1999), leading to endothelial activation and leukocytes attraction (Buckley et al., 2001; Enzerink and Vaheri, 2011).

Interestingly, the impact of microvascular endothelial cells microenvironment on skin integrity in a low-grade inflammatory context is poorly understood. The aim of the present study was to evaluate the impact of the microenvironment produced by dermal microvascular endothelial cells, secondary to a pro-inflammatory challenge, on 2D culture models using dermal fibroblast from healthy donors. We hypothesized that specific endothelial low grade inflammation could change fibroblasts phenotype and be involved in ECM modification and skin alteration. We validated this hypothesis in 2D culture model and then confirmed these results in a 3D full-thickness reconstructed human skin.

## Materials and Methods

### Cell Culture

Human dermal microvascular endothelial cells (HDMEC, Promocell Heidelberg, Germany) were cultured in Endothelial cell medium from Promocell composed by DMEM containing heparin 90 μg/ml, hydrocortisone 1 μg/ml supplemented with 2% Fetal Bovine Serum (FBS), 0.4% Endothelial Cell Growth Supplement (Promocell). Complete medium was supplemented with 1% penicillin/streptomycin (Gibco, Life Technologies, Carlsbad, CA, United States) at 37°c and 5% CO_2_. Human dermal fibroblasts were kindly provided by the Cell and Tissue Bank (Hôpital Edouard-Herriot, Lyon, France). Normal abdominal human skin tissue explants were obtained from the surgical discard of anonymous healthy patients with informed consent of adult donors in accordance with ethical guidelines (French Bioethics law of 2004) and declared to the French research ministry (Declaration no. DC-2008-162 delivered to the Cell and Tissue Bank of Hospices Civils de Lyon). Exclusion criteria were related to positive test result for hepatitis B or C, or HIV as well as obesity history. Human dermal fibroblasts were cultured in a medium consisted in a ratio 1 :1 of Dulbecco’s Modified Eagle’s Medium with Glutamax and F-12 medium (Invitrogen, Cergy Pontoise, France) supplemented with 10% FBS (Sigma-Aldrich, Saint-Quentin-Fallavier, France) and 1% penicillin/streptomycin (Gibco Life Technologies). HDMEC were used below 10 population doubling level and dermal fibroblasts below 20.

### Antibodies and Reagents

The following antibodies were used: Rabbit polyclonal anti-collagen I (Novotec, Bron, France), Rabbit polyclonal anti-elastin antibody (Abcam, Cambridge, Royaume-Uni), Rabbit monoclonal anti-pro-LOX antibody (D8F2K, Cell Signaling), Rabbit polyclonal anti-β-actin antibody (ab8227, Abcam Cambridge, Royaume-Uni). As secondary antibody, Goat anti-rabbit IgG Alexa Fluor 488 (Life Technologies) was used for immunofluorescence and Goat anti-rabbit IgG, HRP-linked antibody for western blot (Cell signaling). Nuclear counterstaining using DAPI was purchased from Sigma-Aldrich. rhIFN-γ (c-60724), rhTNF-α (c-63719), and IL-1β (c-61120) were purchased from Promokine (Heidelberg, Germany).

### Inflammatory Conditioned Medium From HDMEC

To produce a pro-inflammatory CM from HDMEC, cells were seeded in a 75 cm^2^ flask (4 × 10^5^ cells/flask) 24 h before stimulation. HDMEC were stimulated with 0 IU/mL as control, 10 IU/mL or 100 IU/mL (for 3D skin models experiments) of a mix of cytokines composed by rhIFN-γ, rhTNF-α, and rhIL-1β during 24 h at 37°C 5% CO_2_ (CMØ, control; CM10, conditioned medium produce with 10 IU/mL cytokines mix; CM100, conditioned medium produce with 100 IU/mL cytokines mix). Conditioned media were then collected and centrifuged at 10000 ×*g* for 10 min at 4°C. Supernatant of each stimulation experiment was then collected and stored at -80°C until used.

### Effect of the Conditioned Medium on Human Dermal Fibroblasts

Human dermal fibroblasts extracted from abdominal skin biopsies from adult donors were seeded in 6 well plates (200 000 cells/well). 24 h after cell adhesion, fibroblasts medium was changed by endothelial medium during 24 h. Dermal fibroblasts were then washed twice with PBS and stimulated with the different CM obtained (CMØ or CM10) during 48 h at 37°C 5% CO_2_. Results were obtained from 3 independent experiments from 3 donors of fibroblasts.

### Effect of the Conditioned Medium on 3D Human Full Thickness Skin

Full-thickness human 3D skin models (from Lab Skin Creations, Lyon, France) were cultured in 6 well plates using an insert membrane of 0.4 μm of pore size (Millicell, Burlington, United States). Reconstructed skins were cultured in skin medium provided by Lab Skin Creations. 24 h before stimulation, skins were cultured in endothelial cells medium and then stimulated with the different CMs (CMØ or CM100). From preliminary tests it has been established on human biopsies that CM100 and 5 days of stimulation were necessary to observe modifications on skin integrity in presence of CM. 3D Skin models were then stimulated with CM100 during 5 days at 37°C under 5% of CO_2_. Results were obtained from 7 independent experiments using 3D skin models.

### RNA Isolation and Real-Time Quantitative PCR

Total RNA was isolated using RNeasy Kit for 2D *in vitro* models and RNeasy Fibrous Tissue Mini Kit for 3D skin models (Qiagen, Courtaboeuf, France) according to the manufacturer’s instructions. 500 ng of total RNA was reverse-transcribed into cDNA using PrimeScriptTM RT reagent kit (Takara, Shiga, Japan) and analyzed by Real-Time QPCR using SYBR^^®^^ Premix Ex Taq II (Takara) on an AriaMx Realtime PCR system (Agilent Genomics, Santa Clara, CA, United States). All primer (Supplementary Table S1) were provided by Eurogentec. Results were normalized to the *GAPDH* expression level for HDMEC, and dermal fibroblast samples.

Results were obtained from 3 independent experiments and 3 donors of fibroblasts have been used for 2D experiments. Relative quantification was calculated using the 2ΔΔCt quantification method. The primers are listed in the Supplementary Table S1.

### Western Blotting

Dermal fibroblasts were lysed in RIPA buffer from Sigma and supplemented with protease and phosphatase inhibitor at 4°C. Cells lysate was centrifuged 10 min at 10000 *g* and 4°C. Supernatant were collected. Proteins were denatured 5 min at 95°C, separated on 10% polyacrylamide gel according to protein size and transferred to polyvinylidene difluoride (PVDF) membranes (Merck Millipore, Ref. IPVH00010). Membranes were blocked with 1× tris–buffered saline (TBS) 0.1% Tween and 5% non-fat milk for 1 h and blotted at 4°C overnight with primary antibodies diluted as recommended by the manufacturers in 1× TBS, 0.1% Tween and 5% non-fat milk. Membranes were incubated for 1 h at room temperature with secondary antibodies diluted as recommended by the manufacturers in 1× TBS, 0.1% Tween. Then membranes were incubated 5 min with ECL (Thermo Fisher, Ref. 34080) and were revealed by chemiluminescence. Western blot results were analyzed using GelAnalyzer software.

### Immunofluorescence Microscopy

For immunofluorescence, conditioned media (CMØ or CM10) were added when fibroblasts were at confluence on coverslip. Eight-day post confluence, cells were fixed in cold methanol (-20°C) for 20 min to investigate ELN network or were fixed in PBS supplemented with 4% paraformaldehyde at room temperature for 10 min to study collagen I network. Cells were incubated overnight at 4°C with a rabbit polyclonal anti-collagen I (Novotec, Bron, France) antibody diluted at 1:400 in PBS – goat serum 0.1% or a rabbit polyclonal anti-elastin antibody (Abcam, Cambridge, Royaume-Uni) diluted at 1:250 in PBS – goat serum 0.1%, This was followed by a second incubation with a goat anti-rabbit IgG Alexa Fluor 488 (Life Technologies) diluted in 1:1000 PBS – goat serum 0.1% (Life Technologies) at room temperature for 1 h. Nuclear counterstaining using DAPI (Sigma) was carried out with a dilution 1:500 in PBS – goat serum 0.1%. Negative controls were performed by omitting the primary antibody. Image acquisition was carried out using a Nikon microscope (Nikon TE300, Champigny-sur-Marne, France) with a coolsnap fx CCD camera (Photometrics, Tucson, AZ, United States) with MetaVue software (Universal Imaging Corporation, West Chester, PA, United States). Image analysis and semi-quantification were performed using ImageJ software. Results were obtained from 4 independent experiments from 3 donors of fibroblasts. Two images were acquired per condition.

### Luminex Assay

The determination of the amount of multiple cytokines in CMØ and CM10 was carried out using Human Magnetic Luminex Assay from R&D Systems (Minneapolis, MN, United States) according to the manufacturer’s instructions. Levels of CCL2 (MCP-1), IL-6, IL-8 (CXCL8), VEGF-A, IFN-γ, TNF-α, and IL-1β were analyzed using Bio-Plex MAGPIX Multiplex Reader and *Bio-Plex* Manager software from Bio-Rad (Bio-Rad Laboratory, Hercules, CA, United States).

### Statistical Analysis

All statistical analyses were performed using Prism (version 7.0, GraphPad Software Inc.). All results are expressed as mean ± SD. Statistically significant differences were calculated by one-way analysis of variance (followed by Dunnett’s test post-test) or unpaired Student’s *t*-test. *p* < 0.05 was considered statistically significant. ^∗^*P* < 0.05, ^∗∗^*P* < 0.01, ^∗∗∗^*P* < 0.001, ^∗∗∗∗^*P* < 0.0001.

## Results

### HDMEC Activation and Secretion Under Low-Grade Inflammatory Stimulation

**FIGURE 1:**
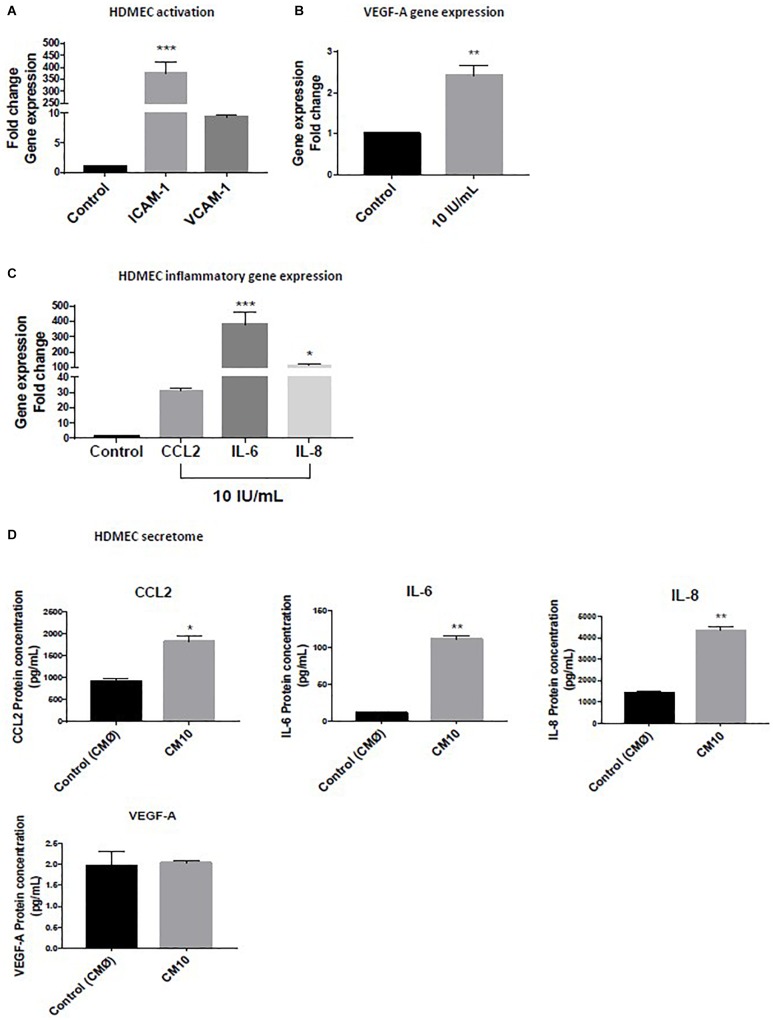
Effect of low grade inflammatory stimulation on HDMEC Gene expression of **(A)** markers of vascular activation of **(B)** *VEGF-A* **(C)** pro-inflammatory cytokines. **(D)** Expression of proteins secreted in the conditioned medium. Luminex assay for CCL2, IL-6, and IL-8 levels and VEGF-A protein expression. Gene expression was normalized with *GAPDH*. Data are representative of at least three independent experiments. Analysis of cytokine secretion was performed in duplicate for each sample using *t*-test. For vascular inflammation results were compared and analyzed to control condition using ANOVA Dunnett’s test. ^∗^*P* < 0.05, ^∗∗^*P* < 0.01, ^∗∗∗^*P* < 0.001.

Human Dermal Endothelial Cells were treated with low-grade inflammatory mix (rhIFN-γ, rhTNF-α, and rhIL-1β) and we observed an upregulation of *ICAM-1* and *VCAM-1* (Figure 1A) demonstrating that the low-grade inflammatory mix activated the dermal microvascular endothelial cells, as confirmed by 2.5-fold increase in *VEGF-A* gene expression (Figure 1B).

We observed a significant HDMEC transcriptional activation of the pro-inflammatory gene expression markers, *CCL2, IL-6*, and *IL-8* (Figure 1C) in response to the initial inflammatory cocktail that resulted of an increased in HDMEC protein secretion of CCL2, Il-6, and IL-8 in the CM10 compared to the control condition CMØ (Figure 1D). The protein fold increases were 1.5, 12, and 2.8, respectively (Figure 1D). In contrast, protein expression of TNF-α, IL-1β, and IFNγ were not detectable in CM10, revealing absence or non-significant levels of these cytokines in HDMEC secretome. VEGF-A protein expression was not significantly different between the two groups (Figure 1D).

### Pro-inflammatory Endothelial Conditioned Medium Stimulates Further Fibroblast Inflammatory Gene Expression

**FIGURE 2:**
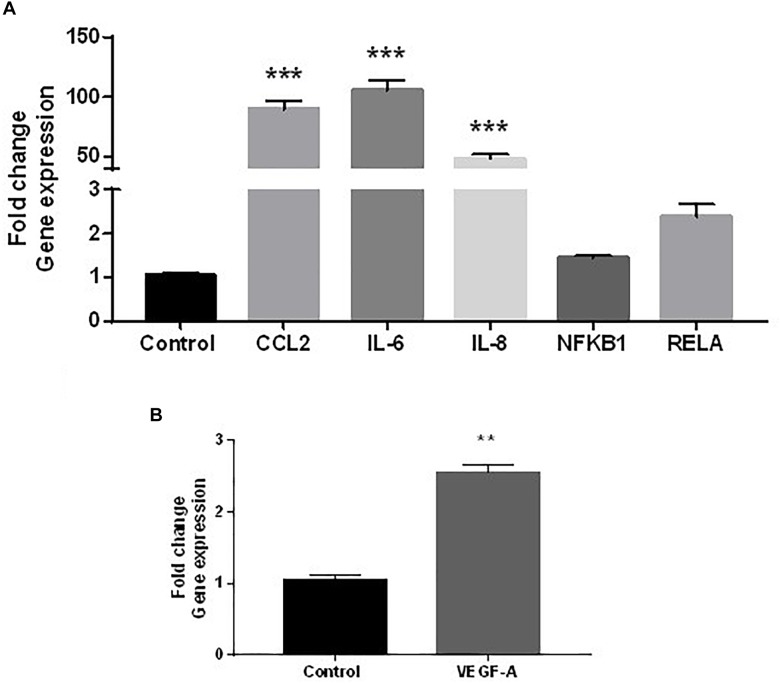
Effect of CM10 on fibroblast gene expression. RT-qPCR expression analysis of genes coding **(A)** *CCL2*, *IL-6*, and *IL-8*, *NFκB1*, *RELA* and **(B)** *VEGF-A* following CM10 stimulation versus control CMØ. Gene expression was normalized with *GAPDH*. **(A)** Results were compared and analyzed to control conditions using ANOVA Dunnett’s test, except for *VEGF-A* where gene expression data were analyzed using a *t*-test. ^∗∗^*P* < 0.01, ^∗∗∗^*P* < 0.001.

The secretion of increased inflammatory cytokines released from HDMEC under low-grade inflammation could influence dermal fibroblasts function. To investigate this possibility in a cell culture system, dermal fibroblasts were stimulated with CMØ or CM10 for 48 h. It appeared that *CCL2*, *IL-6*, and *IL-8* gene expression were significantly up-regulated in dermal fibroblasts in presence of CM10 compared to control condition CMØ (Figure 2A). In contrast, no significant differences were observed in pro-inflammatory NF-κB pathway, NF-κB sub-unit *NFκB1* and *RELA* gene expression in fibroblasts challenged by CM10 compared to CMØ.

*VEGF-A* gene expression was also 2.5-fold increased by dermal fibroblasts under CM10 compared to CMØ (Figure 2B).

### Pro-inflammatory Endothelial Conditioned Medium Altered ECM

**FIGURE 3:**
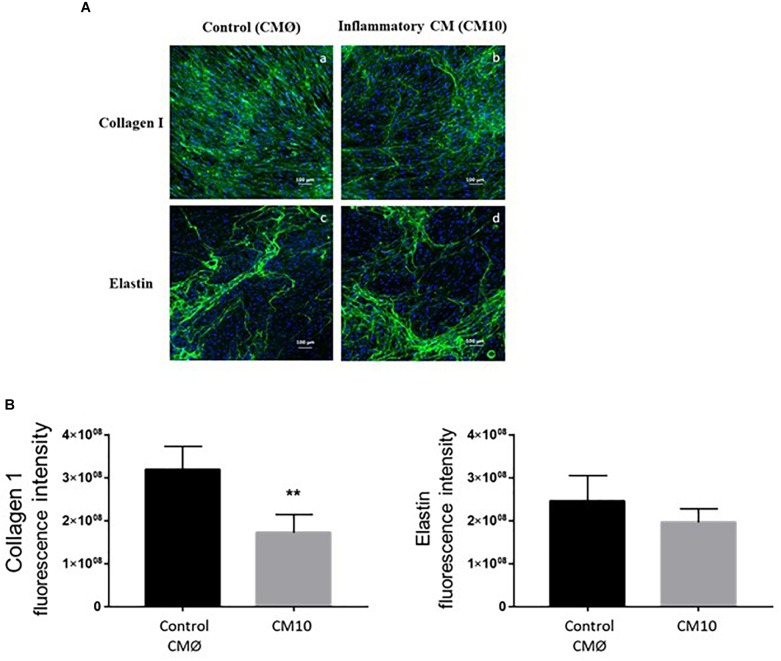
Effect of CM10 on ECM formation. **(A)** Using immunofluorescence assay for ECM formation, collagen I (a,b) and ELN (c,d) were analyzed 8-day post confluence following CM0 or CM10 stimulation. ECM components were stained with anti-collagen1 antibody (a,b) or anti-Elastin antibody (c,d) and fluorescent signals specific to the antibodies were visualized as green. **(B)** Semi-quantification of collagen 1 and ELN positive staining in each condition. Integrated density was used to quantify immunofluorescence. Nuclei were counter-stained with DAPI (blue). Scale bars indicate 100 μm. ^∗∗^*p* < 0.01 vs control.

In order to investigate collagen type I and ELN ECM formation in contact with CMØ or CM10, we used immunofluorescence to analyze their production within the ECM. We confirmed that dermal fibroblasts treated with CM10, 8-day post confluence, synthesized less collagen I than cells treated with CMØ (Figure 3A). Collagen I network appeared to be less organized in presence of CM10 compared to CMØ. Semi-quantification of collagen I labeling was significantly decreased in presence of CM10 compared to CMØ while ELN labeling semi-quantification tended to be reduced but did not reach significant differences (Figure 3B).

**FIGURE 4:**
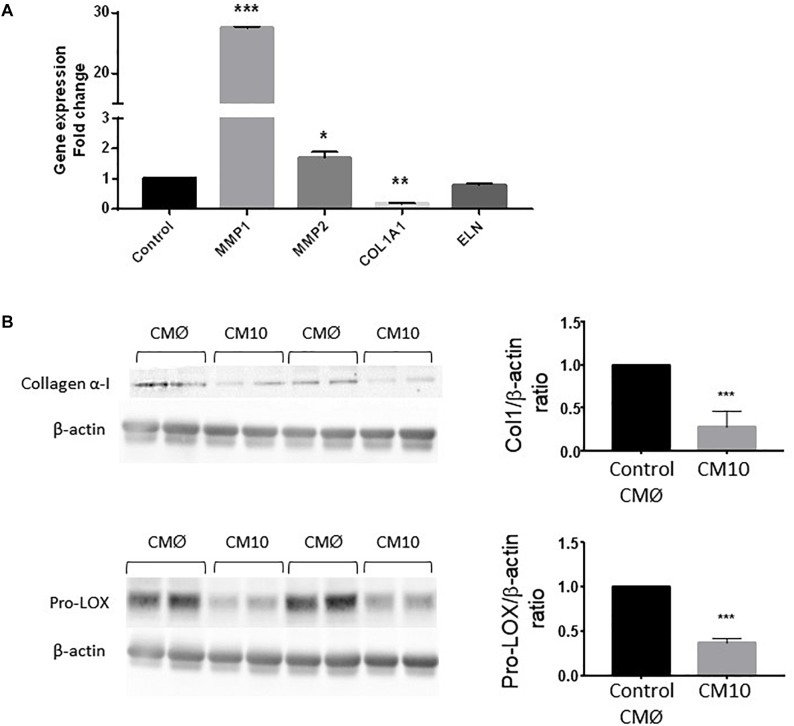
Effect of CM10 on fibroblast ECM gene expression. **(A)** RT-qPCR expression analysis of genes encoding *MMP1*, *COL1A1*, *MMP2*, and *ELN* and **(B)** collagen 1 and pro-LOX protein expression in dermal fibroblasts ECM cultured following CM10 stimulation versus control. Gene expression was normalized with *GAPDH*. Protein expression was normalized using β-actin and quantification was performed using Gel analyzer software. Results were compared and analyzed to control conditions using ANOVA Dunnett’s test, ^∗^*P* < 0.05, ^∗∗^*P* < 0.01, ^∗∗∗^*P* < 0.001.

We then analyzed the expression of genes encoding proteins involved in ECM rearrangement in fibroblasts treated with CM10 or CMØ (Figure 4A). *MMP1* and *MMP2* gene expression were significantly increased by CM10 while *ELN* gene expression was unchanged and *COL1A1* gene expression was significantly reduced. In addition, collagen I protein expression was reduced in presence of CM10 compared to CMØ (Figure 4B) confirming the reduction of the collagen I network as observed by immunofluorescence (Figure 3A). Pro-LOX protein expression, the pro-enzyme form of LOX responsible for collagen and/or ELN cross-link, was also reduced by CM10 compared to CMØ (Figure 4B).

### Effect of CM100 on 3D Human Full Thickness Skin Gene Expression

**FIGURE 5:**
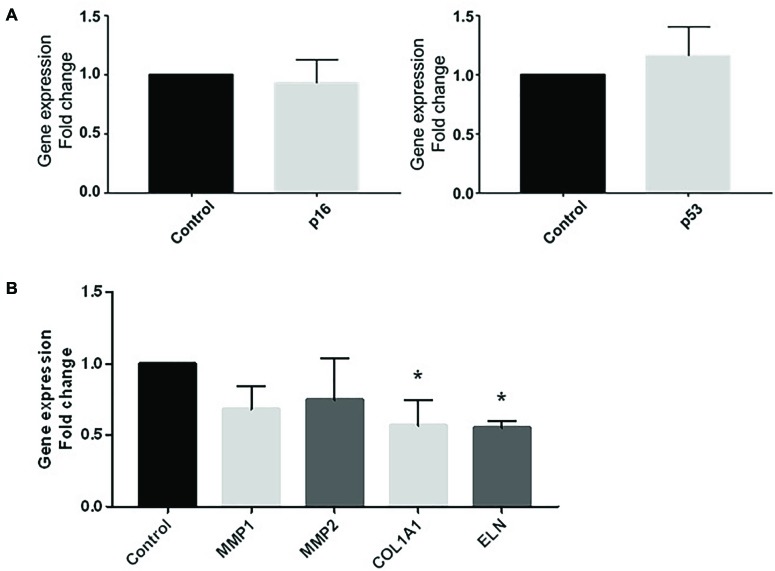
Effect of CM100 on 3D human full-thickness reconstructed skin. RT-qPCR expression analysis of **(A)** stress markers *p16* and *p53* and **(B)** ECM gene *MMP1*, *MMP2, COL1A1*, and *ELN* expression after 5-day stimulation of 3D skin models with CM100 stimulation versus control CMØ. Gene expression was normalized with *GAPDH*. Results were compared and analyzed to control condition using ANOVA Dunnett’s test, ^∗^*P* < 0.05.

To go further into the comprehension of the effect of vascular pro-inflammatory CM on ECM, we used 3D reconstructed skin including keratinocytes and fibroblasts that were challenged with CMØ or CM100 for 5 days. Gene expression of cellular senescence marker *p16* and the stress marker *p53* were both not different between CMØ and CM100 (Figure 5A).

In 3D skin model, *COL1A1* and *ELN* gene expression were significantly downregulated in presence of CM100 (Figure 5B). Nevertheless, in this model there was no upregulation of *MMPs* in presence of CM100 compared to CMØ.

## Discussion

In the present study, we showed that the activated endothelial cell secretome released following pro-inflammatory stress promoted a pro-inflammatory fibroblast phenotype that could be self-sustained without any immune cells. Indeed, CCL2, IL-6, and IL-8 released in the endothelial secretome following IFNγ, TNFα, and IL-1β stress, are responsible for increasing *CCL2, IL-6*, and *IL-8* fibroblast gene expression generating a low-grade inflammatory fibroblast environment. The pro-inflammatory cocktail could thus maintain a perpetual fibroblast inflammatory phenotype that could be responsible for an exacerbation in ECM degradation with an increase in *MMP1* gene expression and unstable collagen 1 network.

Dermal microcirculation is the conduit in which the stressors will affect the surrounding environment. In homeostasis, fibroblasts and endothelial cells are both involved in the angiogenic process and in ECM formation (Slany et al., 2016). In a pro-inflammatory microenvironment, dermal microvascular endothelial cells play an important role in homeostasis and in inflammatory responses. HDMECs synthesize and secrete chemokine and cytokines which are implicated in recruiting immune cells in response to inflammatory disorders (Qazi et al., 2011). This inside-out inflammatory response has been supplemented with an outside-in signaling involving activated pro-inflammatory fibroblasts interacting with immune cells to direct endothelial activation (Enzerink and Vaheri, 2011). In this context, fibroblasts play a critical role in modulating immune cells such as leukocytes and could be responsible for the establishment of chronic inflammation (Buckley et al., 2001).

In the present study, we focused on activated endothelial cell secretion without any immune cell interaction that could impact dermal cell behaviors such as dermal fibroblast and the ECM formation. To play their roles, endothelial cells have to be activated in order to interact with their environment. In an inflammatory context, endothelial cells release different inflammatory factors like IL-1β and TNF-α or by an upregulation of leucocyte adhesion molecule as *ICAM-1* and *VCAM-1*, which are two markers used to confirm the endothelial cells activation (Detmar et al., 1992; Videm and Albrigtsen, 2008). Endothelial cell activation permits the interaction with the environment, playing a key role in inflammatory development (Swerlick et al., 1992). In our models, we showed that HDMEC were activated in presence of low-grade inflammation with an upregulation of *ICAM-1* and *VCAM-1*. These activation are responsible for the loss of vascular integrity allowing secretion of cytokines and chemokines that could interact with tissues like skin structure (Adams and Shaw, 1994). HDMECs are described as critical elements for releasing proteins like VEGF-A or other growth factors. Moreover, HDMECs are also implicated in the cutaneous inflammation, secreting cytokines and chemokines that could directly interact with components of the dermis (Swerlick and Lawley, 1993). It appeared that low-grade inflammatory mix (CM10) composed of 10 IU/mL of IL-1β, TNF-α, and IFN-γ induced upregulation of cytokines and chemokines on dermal microvascular endothelial cells as CCL2, IL-6, and IL-8 which are known to be chemoattractant for T cells, dendritic cells and monocytes. The addition of the activated endothelial secretome onto dermal fibroblasts induced a switch from normal to pro-inflammatory phenotype with an upregulation of *CCL2*, *IL-6*, and *IL-8* gene expression perpetuating fibroblasts pro-inflammatory environment.

The crosstalk between endothelial cells and fibroblasts has been studied through fibroblasts influencing endothelial cells, since fibroblasts provide ECM support as well as the production of several molecules like VEGF for angiogenesis (Villaschi and Nicosia, 1994; Martin et al., 2001; Berthod et al., 2006; Stunova and Vistejnova, 2018). In addition, (Villaschi and Nicosia, 1994) suggested that endothelial cells might also affect fibroblasts since cytokines, chemokines and other molecules present in the microenvironment could affect skin integrity altering ECM components (Van Linthout et al., 2014). In skin aging, it has been shown that IL-8 and IL-6 are clearly linked to MMPs modulation and *COL1A1* gene and protein expression (Dasu et al., 2003; Kolar et al., 2012; Quan and Fisher, 2015). We showed that the low-grade inflammatory microenvironment produced by activated endothelial cells induced a strong up-regulation of *MMP1* in dermal fibroblasts suggesting collagen I degradation associated to a reduction in collagen I synthesis due to *COL1A1* gene downregulation. In addition, the significant decrease in Pro-LOX protein expression strengthens the reduction of collagen cross-linking. Indeed, the primary function of the LOX family is the covalent cross-linking of collagens and/or ELN into a stable, insoluble ECM, which is important for the maintenance of tensile strength and structural integrity of most tissues (Noblesse et al., 2004). The collagenous matrix deposited during LOX reduction should be more easily degradable (Trackman, 2005). In contrast, *ELN* gene expression was not modulated by the vascular pro-inflammatory microenvironment. Only a few studies have evaluated skin ELN production in an inflammatory context apart from skin aging (Ganceviciene et al., 2012) and wound healing (Almine et al., 2013) studies. We confirmed the 2D results with a downregulation of *COL1A1* and the 3D skin model revealed a significant reduction in *ELN* gene expression, suggesting that specific inflammatory vascular environment could alter skin integrity, inducing modification of gene implicated in ECM components. These results show the significant role of skin microvessels on skin integrity, which may vary depending on inflammatory conditions.

Indeed, the loss of vascular integrity is involved in the development of skin or autoimmune diseases, inducing inflammatory processes as chronic inflammatory conditions like psoriasis or lupus (Heidenreich et al., 2009; Achtman and Werth, 2015). Endothelial cells play a significant role in inflammatory responses and link to pro angiogenic and pro-inflammatory protein secretion as described. Among these pro-inflammatory proteins, VEGF-A is described as a predominant protein implicated in immune response. In inflammatory conditions, it appeared that VEGF is not only secreted by endothelial cells but also by macrophages, T cells and fibroblasts, which are involved in angiogenesis and inflammatory processes (Mor et al., 2004; Enzerink and Vaheri, 2011). In our study, it appeared that HDMEC up-regulated *VEGF-A* gene expression, but no differences were observed in VEGF-A protein secretion in the CM, after 24 h of stimulation with the low-grade inflammatory cytokines mix. This result is probably linked to the short biological half-life, and the rapid metabolic degradation of VEGF-A in the medium (Lee et al., 2000).

To conclude, this study showed that the secretory microenvironment related to endothelial inflammation could trigger a perpetual fibroblast inflammatory phenotype that could be responsible for an exacerbation in ECM degradation with an increase in MMP1 and unstable collagen 1 network. In contact with endothelial inflammatory secretion, and no immune cells, it appears that fibroblasts switch from normal to pro-inflammatory phenotype and are less involved in ECM synthesis.

## Author Contributions

LL, GA, JL, GL, and DS-R conceived and designed the study. BS and JD acquired, analyzed, and interpreted the data. BS, LL, JD, GA, JL, GL, and DS-R critically revised the manuscript for important intellectual content. DS-R obtained funding. BS, LL, JD, GA, JL, GL, and DS-R provided the administrative, technical, or material support. DS-R is the guarantor of this work and, as such, had full access to all of the data in the study and takes responsibility for the integrity of the data and the accuracy of the data analysis.

## Conflict of Interest Statement

The authors declare that the research was conducted in the absence of any commercial or financial relationships that could be construed as a potential conflict of interest.

## Supplementary Material

TABLE S1 Details of specific primers and targets used in real-time qPCR experiments.

## Abbreviations

CCL2: chemokine ligand 2
CM: conditioned medium
COL1A1: collagen 1 alpha 1
ECM: extracellular matrix
ELN: elastin
HDMEC: Human Dermal Microvascular Endothelial Cell
ICAM-1: InterCellular Adhesion Molecule 1
IFN-γ: Interferon gamma
IL-1β: Interleukin 1 Beta
IL-6: Interleukin 6
IL-8: Interleukin 8
LOX: Lysyl Oxydase
MMP: Matrix metalloproteinase
pro-LOX: pro-Lysyl Oxydase
TNF-α: Tumor Necrosis Factor alpha
VCAM-1: Vascular cell adhesion protein 1
VEGF-A: Vascular endothelial growth factor A
