# Supplementary material for: Impact of Human Dermal Microvascular Endothelial Cells on Primary Dermal Fibroblasts in Response to Inflammatory Stress
Source: Front Cell Dev Biol. 2019 Apr 3;7:44. doi: 10.3389/fcell.2019.00044 (PMC6456658; doi:10.3389/fcell.2019.00044)
Supplement: TABLE S1 — Details of specific primers and targets used in real-time qPCR experiments. [file Table_1.DOCX]

Supplementary Table

| Gene | Gene ID | Primer | Primer sequence |
| --- | --- | --- | --- |
| *VEGFA* | 7422 | Forward | 5’ – TGC TGT CTT GGG TGC ATT GG – 3’ |
|  |  | Reverse | 5’ – GCA TAA TCT GCA TGG TGA TGT TGG – 3’ |
| *COL1A1* | 1277 | Forward | 5’ – CAG CCG CTT CAC CTA CAG C – 3’ |
|  |  | Reverse | 5’ – TTT TGT ATT CAA TCA CTG TCT TGC C – 3’ |
| *ELN* | 2006 | Forward | 5’ – GTT GGT GGC TTA GGA GTG TCT G – 3’ |
|  |  | Reverse | 5’ – CGG CAC TTT CCC AGG CTT C – 3’ |
| *MMP1* | 4312 | Forward | 5’ – GCT TTC CTC CAC TGC TGC T – 3’ |
|  |  | Reverse | 5’ – CTT GCC TCC CAT CAT TCT TC – 3’ |
| *MMP2* | 4313 | Forward | 5’ – TCT TCC CCT TCA CTT TCC TG – 3’ |
|  |  | Reverse | 5’ – ACT TGC GGT CGT CAT CGT – 3’ |
| *ICAM1* | 3383 | Forward | 5′-AGG CCA CCC CAG AGG ACA AC-3′ |
|  |  | Reverse | 5′- CCC ATT ATG ACT GCG GCT GCT A-3′ |
| *VCAM1* | 7412 | Forward | 5′-CGT CTT GGT CAG CCC TTC CT-3′ |
|  |  | Reverse | 5′-ACA TTC ATA TAC TCC CGC ATC CTT C-3′ |
| *IL6* | 3569 | Forward | 5′-GAA CTC CTT CTC CAC AAG CGC CTT-3′ |
|  |  | Reverse | 5′-CAA AAG ACC AGT GAT GAT TTT CAC CAG G-3′ |
| *IL8* | 3576 | Forward | 5′-TCT GCA GCT CTG TGT GAA GG-3′ |
|  |  | Reverse | 5′-ACT TCT CCA CAA CCC TCT GC-3′ |
| *CCL2* | 6347 | Forward | 5′-AGC AAG TGT CCC AAA GAA GC-3′ |
|  |  | Reverse | 5′-CAT GGA ATC CTG AAC CCA CT-3′ |
| *NFKB1* | 4790 | Forward | 5′-GCA GAT GGC CCA TAC CTT CA-3′ |
|  |  | Reverse | 5′-CAC CAT GTC CTT GGG TCC AG-3′ |
| *RELA* | 5970 | Forward | 5′-CCA GAC CAA CAA CAA CCC CT-3′ |
|  |  | Reverse | 5′-TCA CTC GGC AGA TCT TGA GC-3′ |
| *GAPDH* | 2597 | Forward | 5’-CCG GGA AAC TGT GGC GTG ATG G-3’ |
|  |  | Reverse | 5’-AGG TGG AGG AGT GGG TGT CGC TGT T-3’ |
| p16 | 1029 | Forward | 5’-CCA ACG CAC CGA ATA GTT ACG– 3’ |
|  |  | Reverse | 5’-GCT ACC TGA TTC CAA TTC CCC T– 3’ |
| P53 | 7157 | Forward | 5’-AGT ATT TGG ATG ACA GAA– 3’ |
|  |  | Reverse | 5’-ATG TAG TTG TAG TGG ATG– 3’ |
